# Empagliflozin repurposing in Parkinson’s disease; modulation of oxidative stress, neuroinflammation, AMPK/SIRT-1/PGC-1α, and wnt/β-catenin pathways

**DOI:** 10.1007/s10787-023-01384-w

**Published:** 2023-12-01

**Authors:** Noha Nabil Mohammed, Mariane G. Tadros, Mina Y. George

**Affiliations:** Department of Pharmacology and Toxicology, Faculty of Pharmacy, Ain Shams University, Cairo Governorate, 11566, Egypt

**Keywords:** Empagliflozin, Parkinson's disease, AMPK/SIRT-1/PGC*-*1α, Dopamine turnover, Wnt/β-catenin, Inflammation

## Abstract

Parkinson's disease is a neuroprogressive disorder characterized by loss of dopaminergic neurons in substantia nigra pars compacta. Empagliflozin (EMPA), a SGLT-2 inhibitor, is an oral hypoglycemic agent with reported anti-inflammatory and antioxidant effects. The current study aimed to evaluate the neuroprotective effect of EMPA in rotenone-induced Parkinson's disease. Rats were randomly distributed among five groups as follows: control, rotenone (2 mg/kg), rotenone + EMPA (10 mg/kg), rotenone + EMPA (20 mg/kg), and EMPA (20 mg/kg) groups. They were treated for 30 consecutive days. Rotenone reduced locomotor activity and retention time on the rotarod performance test while elongated descent latency time. On the other side, EMPA corrected these behavioral changes. These results were confirmed by histological examination and number of intact neurons. Moreover, rotenone induced alpha-synuclein accumulation, reduced tyrosine hydroxylase expression, dopamine, 3,4-dihydroxyphenylacetic acid, and homovanillic acid concentrations. On the other side, EMPA reversed such effects induced by rotenone. Depending on previous results, EMPA (20 mg/kg) was selected for further mechanistic studies. Rotenone ameliorated superoxide dismutase and catalase activities and enhanced lipid peroxidation, interleukin-1β, and tumor necrosis factor-α levels. By contrast, EMPA opposed rotenone-induced effects on oxidative stress and inflammation. Besides, rotenone reduced the expression of pAMP-activated protein kinase (pAMPK), peroxisome proliferator-activated receptor-gamma coactivator-1α (PGC-1α), and Sirtuin-1 (SIRT-1), as well as abrogated NAD^+^/NADH ratio. However, EMPA activated the AMPK/SIRT-1/PGC*-*1α pathway. Moreover, rotenone hindered the wnt/β-catenin pathway by reducing the wnt-3a level and β-catenin expression. On the other side, EMPA triggered activation of the wnt/β-catenin pathway. Collectively, EMPA may provide a promising solution for Parkinson's patients worldwide.

## Introduction

Parkinson's disease (PD) is the second most common neurodegenerative disorder influencing nearly 1% of people over 60 globally (Wang et al. 2015). Such progressive disorder may result from the gradual loss of dopaminergic neurons in substantia nigra pars compacta. Dopamine (DA) is the responsible neurotransmitter for signal transmission, controlling smooth muscle contraction and movement (Jamwal and Kumar 2019). In addition, PD is associated with the accumulation of alpha-synuclein forming Lewy bodies aggregates. The main features of PD include bradykinesia, resting tremors, rigidity, and postural instability (Stoker and Greenland 2018).

Rotenone, a lipophilic naturally occurring compound, is widely used as a pesticide and piscicide (Heinz et al. 2017). Rotenone acts as a mitochondrial Complex I inhibitor, interfering with the electron transport chain, causing ATP depletion, and enhancing mitochondrial reactive oxygen species (ROS) production (Li et al. 2003). Via selective nigrostriatal dopaminergic degeneration, rotenone may help induce alpha-synuclein inclusion forming Lewy bodies, PD-associated neurobehavioral changes and motor abnormalities, oxidative stress, and inflammatory response (Alam and Schmidt 2002; Sherer et al. 2003).

Oxidative stress was reported to be important in PD pathogenesis (Percário et al. 2020). Oxidative stress may result from free radicals production and ameliorated antioxidant defense mechanisms, resulting in mitochondrial dysfunction and cell death. It was previously reported that PD patients have lowered superoxide dismutase (SOD), catalase (CAT), and glutathione peroxidase activities. Besides, PD was reported to be associated with improved cytokines release and neuroinflammation (Troncoso-Escudero et al. 2018). In addition, microglia and astrocytes secrete cytokines, which are multi-functional immunoregulatory proteins, including tumor necrosis factor-α (TNF-α) and interleukin-1β (IL-1β) (Rasheed et al. 2021). Such enhanced neuroinflammation may result in alpha-synuclein accumulation (Gelders et al. 2018).

Energy metabolism was reported to be highly related to mitochondrial biogenesis. Two metabolic sensors of decreased energy level: Sirtuin-1 (SIRT-1) and AMP-activated protein kinase (AMPK). Both could directly affect the activity of the master regulator peroxisome proliferator-activated receptor gamma co-activator 1-alpha (PGC-1α) via deacetylation and phosphorylation (Shi et al. 2018). The AMPK/SIRT-1/PGC-1α axis may be considered a cornerstone of mitochondrial biogenesis that regulates energy metabolism. Energy depletion, calorie restriction, DNA damage, and neuronal excitatory induced such a pathway (Cantó and Auwerx 2009).

Moreover, wnt/β-catenin signaling is a conserved pathway vital to homeostasis and nervous system development. Additionally, it was observed that dysregulation of such pathways is associated with neuronal dysfunction and death (Marchetti 2018). Therefore, this signaling pathway has been suggested as a potential therapeutic target against neurodegeneration (Serafino et al. 2020).

Empagliflozin (EMPA), a sodium-glucose cotransporter-2 (SGLT-2) inhibitor, was approved to treat type II diabetes mellitus. EMPA was reported previously for its antioxidant (Abed et al. 2020), anti-inflammatory (Amin et al. 2020), and neuroprotective (Mousa et al. 2023) activities. Therefore, the present study aimed to elucidate the neuroprotective effects of EMPA on PD induced by rotenone in rats and contemplate the possible underlying mechanisms.

## Materials and methods

### Animals

Male Wistar rats (10 weeks old, weighing 200–250 g) were purchased from Nile Co. for pharmaceutical and chemical industries in Cairo, Egypt. Animal Handling and experimentation were conducted under ARRIVE guidelines concerning the care and use of laboratory animals. The study was approved by the research ethical committee of the Faculty of Pharmacy, Ain Shams University (Approval No. #REC 163). The animals were left to acclimatize for two weeks before treatment. They were housed in an air-conditioned atmosphere with 12 h light–dark cycles, reared on a balanced laboratory diet, and given water ad libitum.

### Drugs and chemicals

EMPA was purchased from El-Hikma Pharmaceutical Co., Giza, Egypt. Rotenone was purchased from Sigma Aldrich, USA. For Western blot analysis, anti-β-actin (Catalogue No. #ab8224), anti-AMPK (Catalogue No. #ab110036), anti-SIRT-1 (Catalogue No. #ab110304), and anti-β-catenin (Catalogue No. #ab32572) antibodies were purchased from Abcam, USA. Anti-pAMPK (Thr172) (Catalogue No. #2531) anti-PGC-1α (Catalogue No. #2178) antibodies were purchased from Cell Signaling Technology, USA. Anti-tyrosine hydroxylase antibody (Catalogue No. #NB300-109) was purchased from Novus Biological, USA. Anti-alpha-synuclein antibody (Catalog No.# sc-12767) was purchased from Santa Cruz Biotechnology, Inc.

### Experimental design

#### Preliminary dose–response study

Forty rats were randomly distributed among five groups and treated for thirty days as follows: Group I (negative control group) received subcutaneous sunflower oil, as well as 1% dimethyl sulfoxide (DMSO) and polyethylene glycol (PEG) (1:1 v/v) orally daily for 30 days. Group II received rotenone (2 mg/kg, subcutaneous) and 1% oral DMSO and PEG one hour later daily. Group III and IV received rotenone (2 mg/kg, subcutaneously) once daily for 30 days. One hour later, they received EMPA daily for 30 days at doses of 10 mg/kg and 20 mg/kg (oral), respectively. Group V received EMPA 20 mg/kg via oral gavage once daily for thirty days. EMPA and rotenone doses were selected based on previous studies (Amin et al. 2020; Lee et al. 2019). Afterward, rats were subjected to behavioral tests to assess motor function: locomotor activity, grid and bar catalepsy, and rotarod tests. Then, animals were sacrificed after anesthesia (using ketamine), skulls were split on ice, and striata and the midbrains were dissected. Samples from all groups were fixed in 10% buffered formalin and embedded in paraffin for histological examination and toluidine blue staining. In addition, the midbrains and striata samples were immediately immersed in liquid nitrogen and stored at − 80 °C for alpha-synuclein and tyrosine hydroxylase (TH) western blot analysis. Moreover, specimens were homogenized at 1:10 (w/v) in potassium phosphate buffer (pH = 7.5) for biochemical detection of DA, 4-dihydroxyphenylacetic acid (DOPAC), and homovanillic acid (HVA).

#### Mechanistic study

Based on a previous preliminary study, a higher dose of EMPA (20 mg/kg) was selected for further mechanistic studies. Twenty-eight rats were assigned into four groups and treated for thirty days: Groups I and II were treated as previously mentioned. Group III received rotenone (2 mg/kg, subcutaneous) once daily. One hour later, they received EMPA (20 mg/kg, oral) daily. Group IV received oral EMPA (20 mg/kg) alone once daily. Then, as previously mentioned, the striata and the midbrains from all groups were dissected. Samples were immediately immersed in liquid nitrogen and stored at 80 °C for western blot analysis. Other specimens were homogenized at 1:10 (w/v) in potassium phosphate buffer (pH = 7.5) for further biochemical analysis.

### Behavioral assessment

#### Locomotor activity

Opto-Varimex–Mini Model B (Columbus, OH, USA) was used to assess the animal's locomotor activity. It consists of 15 infrared beams (875 nm wavelength and 0.32 cm diameter) located at a distance of 5 cm. IR beams were interrupted by rats' movements, which were sensed and counted. Results were expressed as counts per 5 min (Awad et al. 2023).

#### Catalepsy tests (Grid and Bar tests)

Twenty-four hours after the last treatment, grid, and bar tests tested animals muscle rigidity. Concerning the grid test, rats were placed with their four paws at the center of a 12 × 12 cm mesh grid with 0.5 × 0.5 cm grid openings placed 20 c above the surface. The time required by each rat to descend was determined as descent latency (Zhang et al. 2017). For the bar test, rats were placed gently on a bar (10 cm above the surface) where they held the bar with their forepaws. The time required by each rat to remove one or both paws was recorded. The cut-off time for both tests was 30 s (Neely et al. 2013).

#### Rotarod performance test

Rats ' motor coordination was detected using a rotarod instrument (model 3375-R4, TSE systems) with revolving bars. Rats were subjected to training and testing sessions. The training session continued for 60 s at 4 rpm. During the testing session, the revolving bars' speed was increased from 4 to 40 rpm within 2 min. The time latency for each rat to fall was detected. Each rat was tested in triplicate sessions. The cut-off time was 120 s (Desouky et al. 2023; Matt et al. 2018).

### Histological examination

The midbrains, striata, and substantia nigra from different groups were fixed in 10% neutral buffered formalin for 24 h, then washing and serial dilutions of alcohols were applied for dehydration. In addition, samples were paraffinized at 56 °C for 24 h. Those tissue blocks were cut by slide microtome at 4 μm thickness; the brain sections were collaged on glass slides to be deparaffinized and stained using hematoxylin and eosin. Then, the tissue sections were visualized by a Full HD microscope camera operated by a Leica application module for analysis (Leica Microsystems GmbH, Wetzlar, Germany) (Bancroft and Gamble 2008).

### Intact cell count

An intact cell count was performed using toluidine blue staining. Multiple fields per section were used for intact cell quantitation in the midbrains, striata, and substantia nigra in different groups. Glass slides were visualized using a Full HD microscopic camera operated by a Leica application module for tissue section analysis (Leica Microsystems GmbH, Wetzlar, Germany) (Ibrahim et al. 2021).

### Dopamine turnover

Dopamine turnover was determined by detecting concentrations of DA and its metabolites. DA, DOPAC, and HVA levels were assigned using high-performance liquid chromatography (HPLC) with an electrochemical detector. The neurotransmitter's concentrations were detected using the integration of chromatographic peak areas and expressed as ng/mg protein (Chakraborty et al. 2019).

### Oxidative stress markers

Oxidative stress markers were detected using kits purchased from Biodiagnostics, Giza, Egypt. Antioxidant CAT level in homogenates was estimated according to the method of Aebi (1984). Results were expressed as U/mg protein. Assessment of lipid peroxidation was determined in terms of thiobarbituric acid reactive substances measured as malondialdehyde (MDA). It was determined colorimetrically by Satoh et al. (1981). The results were expressed as nmol/mg protein using 1,1,3,3-tetraethoxypropane as standard. The activity of SOD was detected according to Nishikimi et al. (1972). Briefly, SOD in the midbrains and striata hindered nitroblue tetrazolium dye reduction by phenazine methosulphate. This generates a color determined at 560 nm at time intervals over 5 min. Results were expressed as U/min/mg protein.

### Inflammatory markers

Levels of TNF-α and IL-1β were determined using ELISA kits purchased from Biovision laboratories (Catalogue No. #In-Ra1344 and #In-Ra0668, respectively). Using the ELSIA technique, color intensities were measured at 450 nm using a microplate reader (ChroMate-4300, FL, USA). The quantities of TNF-α and IL-1β were expressed as pg/mg protein.

#### Western blotting of alpha-synuclein, tyrosine hydroxylase, AMPK, pAMPK, SIRT-1, PGC-1α, and β-catenin

The midbrain and the striatum tissue lysates were prepared in RIPA buffer. Protein extraction from supernatants was performed using a ReadyPrepTM protein extraction kit (Catalogue No. #163–2086, Bio-Rad Inc.). Protein was determined in samples using the Bradford Protein Assay Kit (Catalogue No. #SK3041, Markham, Ontario, Canada). Equal protein concentrations from all samples were loaded with sample buffer 4% SDS, 10% 2-mercaptoethanol, 20% glycerol, 0.004% bromophenol blue, and 0.125 M Tris HCl. After boiling for 5 min, samples were loaded on polyacrylamide gel (SDS-PAGE) (Catalogue No. #161–0181, Bio-Rad Inc). A sandwich of PVD and gel was transferred with transfer buffer (25 mM Tris, 190 mM glycine, and 20% methanol), allowing transfer from the gel to the membrane using the BioRad Trans-Blot Turbo apparatus. After membrane blocking for 1 h, incubation with primary antibodies was performed at 4 °C. Afterwards, the solution was added to the HRP-conjugated secondary antibody (Novus Biologicals, USA). Then, the ECL chemiluminescent substrate (Catalogue No.# 170–5060, Bio-Rad, Inc.) was added. The chemiluminescence was captured, and image analysis was performed against β-actin using a ChemiDoc MP imager.

#### NAD^+^/NADH ratio

NAD/NADH levels were determined according to the manufacturer's protocol (Catalogue No. #ab65348, Abcam, USA). Total NAD^+^, NADH, and NAD/NADH ratio were determined at 405 nm, and results were expressed as mg/mg protein.

#### Wnt 3a determination

Wnt 3a concentration was detected using an Enzyme-linked immunosorbent assay kit (Catalogue No. # MBS1608561, MyBioSource, USA) using a wavelength of 450 nm. Results were expressed as ng/mg protein.

#### Detection of total protein

Protein concentration was determined using the Bradford Protein Assay kit (Catalogue No. #SK3041, Ontario, Canada) using BSA as a protein standard.

#### Statistical analysis

Data were presented as mean ± SD. Multiple comparisons were performed for all results using one-way ANOVA followed by Tukey as a post-hoc test. The significance level used was 0.05 level of probability. All statistical analyses were performed using GraphPad Prism software version 7 (GraphPad Software, Inc., La Jolla, CA, USA).

## Results

### Effect of EMPA treatment on behavioral assessment in rotenone-induced PD in rats

#### Locomotor activity

**Fig. 1:**
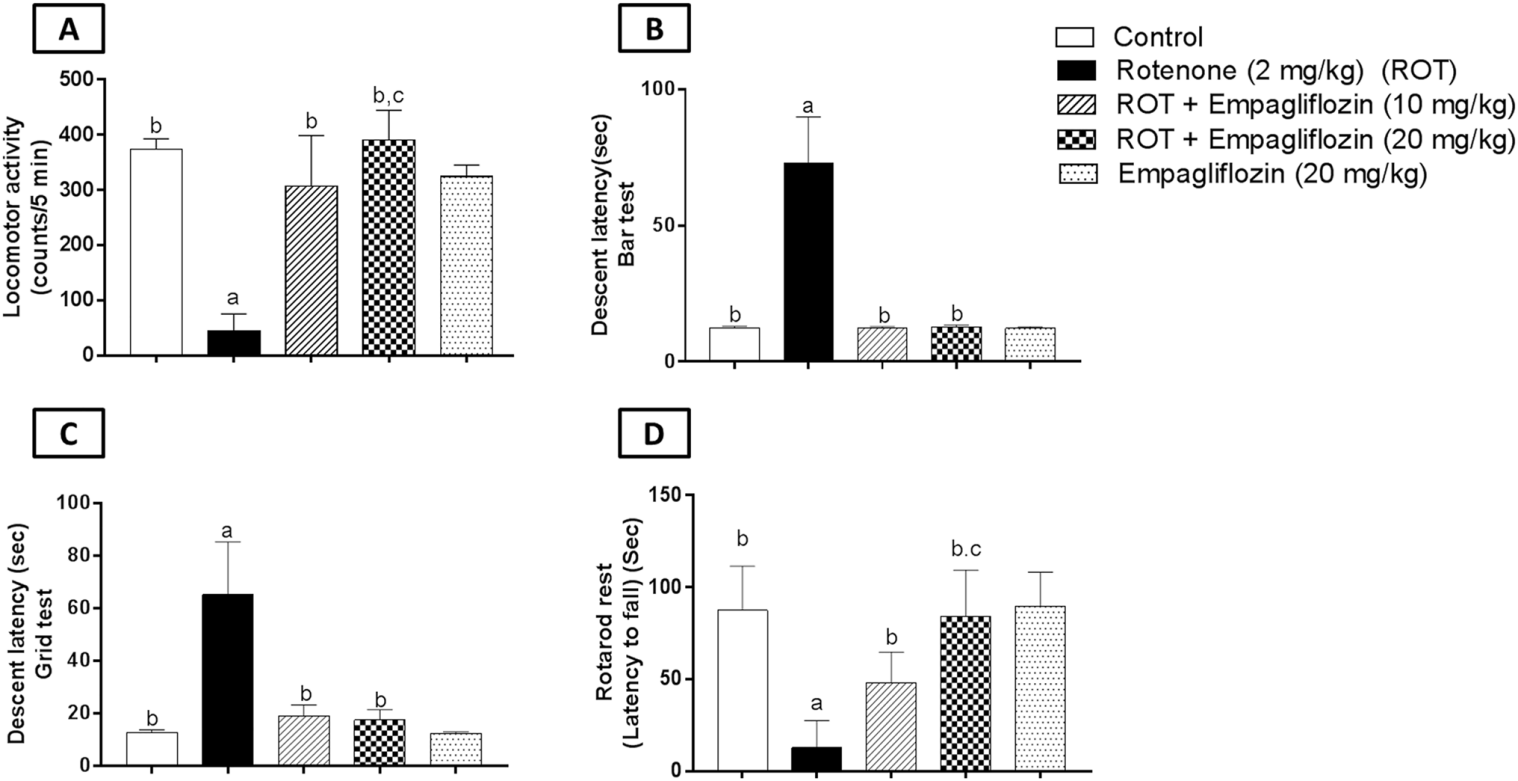
Effect of EMPA treatment on locomotor activity (**A**), descent latency (Bar test) (**B**) descent latency (Grid test) (**C**), and rotarod test (**D**) against rotenone-induced PD in rats. Data are presented as mean ± SD (*n* = 8), where a, b, and c are statistically significant from the control group, rotenone-treated group, and EMPA (10 mg/kg)-treated group, respectively, at *p* < 0.05 using one-way ANOVA followed by Tukey as a post-hoc test

The rotenone-treated group showed a significant decrease in locomotor activity by 5.89 folds compared to the control group (F_4,35_ = 94.28, *p* < 0.0001). On the other hand, both treated groups with EMPA, 10 mg/kg and 20 mg/kg, showed a significant elevation in locomotor activity relative to the rotenone-treated group by 5.96 and 7.75 folds, respectively. Interestingly, the EMPA (20 mg/kg)-treated group showed a significant elevation in locomotor activity by 1.27 folds compared with the EMPA (10 mg/kg) treated group (Fig. 1A).

#### Catalepsy tests (grid and bar tests)

The bar test showed a significantly longer descent time in the rotenone-induced group by 28.4 folds compared to the negative control group (F_4,35_ = 45.87, *p* < 0.0001). Furthermore, EMPA (10 mg/kg)-treated rats exhibited a significantly shorter descent latency period by 30.1 folds compared to the disease group, while 20 mg/kg EMPA treatment illustrated a significant reduction as compared to the rotenone-treated group by 25.2 folds (Fig. 1B).

Additionally, grid test results revealed that rotenone treatment significantly elevates descent latency compared to the vehicle group by 5.13 folds (F_4,35_ = 97.68, *p* < 0.0001). Otherwise, the treated groups with EMPA, either 10 mg/kg or 20 mg/kg, showed a significant decrease by 3.43 folds and 3.72 folds, respectively, relative to the rotenone-treated group (Fig. 1C).

#### Rotarod performance test

One-way ANOVA revealed a significant decline in retention time on comparing the rotenone group with the control group by 6.8 folds (F_4,35_ = 25.78, *p* < 0.0001). While the EMPA (10 mg/kg)-treated group significantly increased the retention time by 3.75 folds. EMPA (20 mg/kg)-treated group presented a significant elevation by 5.18 folds compared to the rotenone-induced group. Interestingly, the EMPA (20 mg/kg)-treated group demonstrated a significant elevation by 1.38 folds compared to the EMPA (10 mg/kg)-treated group **(**Fig. 1D**)**.

### Effect of EMPA treatment on histological examination in rotenone-induced PD in rats

**Fig. 2:**
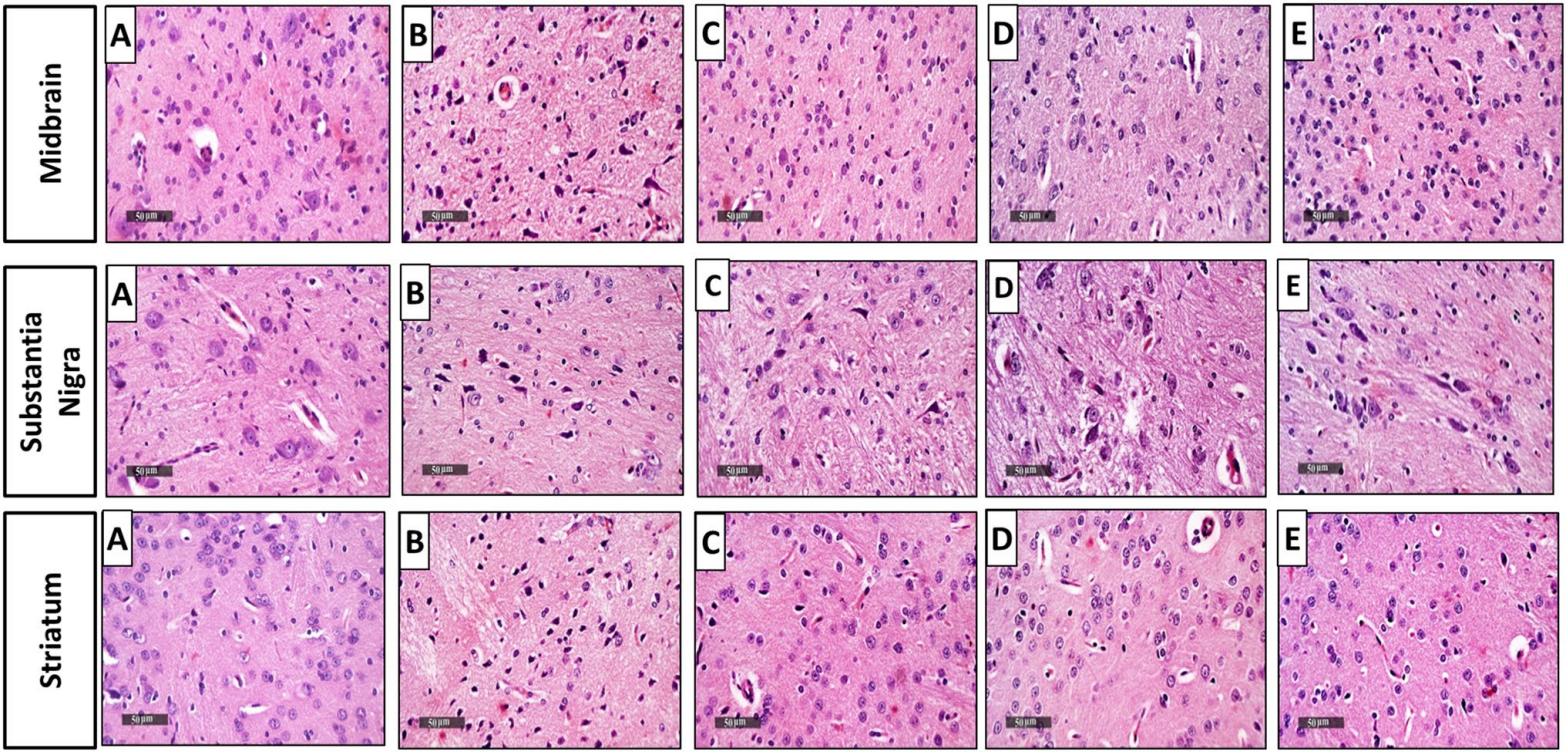
Effects of EMPA treatment on rotenone-induced histological alterations of rats in the midbrain, substantia nigra, and the striatum regions (*n* = 3) (scale bar 50 µm). Photomicrographs of hematoxylin and eosin-stained sections from the control group (**A**); rotenone-treated group (2 mg/kg) (**B**); rotenone and EMPA group (10 mg/kg) (**C**); rotenone and EMPA group (20 mg/kg) (**D**); EMPA alone group (20 mg/kg) (**E**); with 100 × magnification power

Microscopic examination of the midbrain (inferior colliculus subregion) of rats revealed the following: apparent intact well-organized neurons and intact intercellular brain matrix with minimal records of reactive glial cell infiltrates were found in the control group (Fig. 2A). Rotenone-treated rats demonstrated severe neuronal loss and degenerative changes with marked records of pyknotic degenerated neurons and moderate edema of brain matrix accompanied with many reactive glial cells infiltrates (Fig. 2B). Moreover, EMPA (10 mg/kg)-treated group exhibited minimal records of degenerated neurons together with intact intercellular brain matrix (Fig. 2C). Group of rats treated with EMPA (20 mg/kg) showed apparent intact neurons without a significant abnormal histological change (Fig. 2D). Drug alone treated rats showed almost the same features as standard control samples without abnormal histological abnormalities (Fig. 2E).

Concerning substantia nigra pars compacta, control group showed normal histological organization of SN pars compacta with many apparent well-organized intact neurons bearing large vesicular, spherical nuclei, and granular basophilic cytoplasm with intact surrounding intercellular brain matrix **(**Fig. 2A**)**. However, rotenone treatment presented a significant record of neuronal loss and many shrunken and dark eosinophilic necrotic neurons with moderate perineuronal edema. Vacuolization of the brain matrix with significant reactive microglial, astrocytic infiltrates, and congested vasculatures was also detected (Fig. 2B). Treatment with low dose EMPA demonstrated moderate neuroprotective efficacy evidenced by apparent intact neurons with almost intact subcellular details. In addition, damaged and degenerated neurons with the persistence of mild glial cell infiltrates as well as edema were also found (Fig. 2C). The group of rats treated with EMPA at 20 mg/kg showed higher neuroprotective efficiency with minimal degenerative neuronal changes and abundant higher records of apparent intact cell bodies. Minimal abnormal reactive glial cell infiltrates were also shown (Fig. 2D). The alone-treated group illustrated nearly intact morphological features of SN pars compacta (Fig. 2E).

Regarding the striatal region, control group rats revealed regular morphological features of the striatum with many apparent intact neurons of different sizes with intact subcellular and nuclear details. An intact intercellular brain matrix was shown with minimal records of reactive glial cell infiltrates (Fig. 2A). The disease-induced group demonstrated moderate focal areas of darkly stained and pyknotic degenerated neurons with significant neuronal loss and moderate edema of the brain matrix. Also, many reactive microglial cells and reactive astrocytic infiltrates all over striatal regions were detected (Fig. 2B). EMPA (10 mg/kg)-treated rats showed a significant neuroprotective efficacy with scattered few records of degenerated neurons and higher figures of apparent intact neurons. Intact intercellular brain matrix with significantly fewer reactive glial cell infiltrates were observed (Fig. 2C). Higher dose EMPA treatment exhibited almost regular histological features as control samples (Fig. 2D). The group of rats treated with EMPA alone demonstrated typical features of striatal regions without abnormal histological changes (Fig. 2E). Scoring of histological alterations found was added in Table 1.

**Table 1:** Effect of empagliflozin administration on histological alterations induced by rotenone in rats

Area	Group	Neurodegeneration	Pyknosis	Edema	Microglial/ astrocytic cell infiltrates	Congested vasculature
The midbrain	A	−	−	−	−	−
	B	+ + +	+ + +	+ +	+ + +	−
	C	+	+	−	+	−
	D	−	−	−	−	−
	E	−	−	−	−	−
Substantia Nigra	A	−	−	−	−	−
	B	+ + +	−	+ +	+ + +	+ +
	C	+ +	−	+	+	−
	D	+	−	−	+	−
	E	−	−	−	−	−
The striatum	A	−	−	−	−	−
	B	+ +	+ +	−	+ +	−
	C	+	−	−	+	−
	D	−	−	−	−	−
	E	−	−	−	−	−

Effect of EMPA on histological alterations induced by rotenone in a rat model of PD. Rotenone was administered once daily in a dose of 2 mg/kg, s.c., for 30 consecutive days. EMPA was given once daily in doses of 10 and 20 mg/kg orally for 30 consecutive days. Grading of histological alterations was determined as follows: (−) indicates normal histological structure of the midbrain, substantia nigra, and the striatum. (+) indicates mild histological changes of the midbrain, substantia nigra, and the striatum (< 25%). (+ +) indicates moderate histological changes of the midbrain, substantia nigra, and the striatum (< 50%). (+ + +) indicates severe histological changes of the midbrain, substantia nigra, and the striatum changes (< 75%)

Rats were distributed into five groups as follows: (A) indicates the control group, (B) indicates the rotenone-induced group, (C) indicates EMPA (10 mg/kg)-treated group, (D) indicates EMPA (20 mg/kg)-treated group, (E) indicates drug alone group

### Effect of EMPA treatment on intact neurons in rotenone-induced PD in rats

**Fig. 3:**
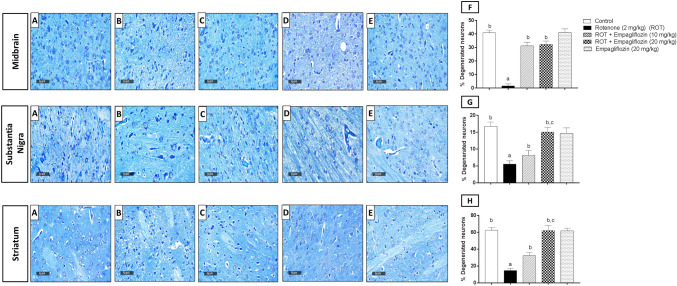
Effect of EMPA treatment on rotenone-mediated neurodegenerative changes using toluidine blue staining. Representative photomicrographs for the control group (**A**); rotenone-treated group (2 mg/kg) (**B**); rotenone and EMPA group (10 mg/kg) (**C**); rotenone and EMPA group (20 mg/kg) (**D**); EMPA alone group (20 mg/kg) (**E**); of stained sections the midbrain, substantia nigra and striata regions (*n* = 3) (scale bar 50 µm). Quantitative determination of intact neurons in the midbrain (**F**), substantia nigra region (**G**), and striata (**H**) across six different fields per rat section for three rats per group. Data are presented as mean ± SD a, b, and c; statistically significant from the control group, rotenone-treated group, EMPA (10 mg/kg)-treated group, respectively, at *p* < 0.05 using one way ANOVA followed by Tukey as a post-hoc test

As shown in Fig. 3, stained midbrains (F_4,25_ = 307.4, *p* < 0.0001), substantia nigra (F_4,25_ = 72.58, *p* < 0.0001), and striata (F_4,25_ = 222.8, *p* < 0.0001) showed a significant increase in degenerated neurons of rotenone-treated rats compared to the control group by 27.22, 3, and 4.29 folds, respectively (Fig. 3F, 3G, and 3H). Conversely, the midbrain stained area showed neuronal degenerative reduction after treatment with the two doses of EMPA, 10 mg/kg and 20 mg/kg, by 20.78 and 21.44 folds, respectively. In the substantia nigra, a significant elevation was observed when comparing the two doses of EMPA, 10 mg/kg and 20 mg/kg, by 1.48 and 2.72 folds, respectively, to the rotenone-induced group. The striatum showed a significant elevation by 2.22 folds on comparing EMPA 10 mg/kg to the disease group and by 4.26 folds on comparing the higher dose of EMPA to group 2. Treatment with the higher dose of EMPA (20 mg/kg) showed a significant reduction in neuronal degeneration compared to the lower dose in substantia nigra and the striatum by 1.83 and 1.91 folds, respectively.

### Effect of EMPA treatment on DA turnover in rotenone-induced PD in rat

**Fig. 4:**
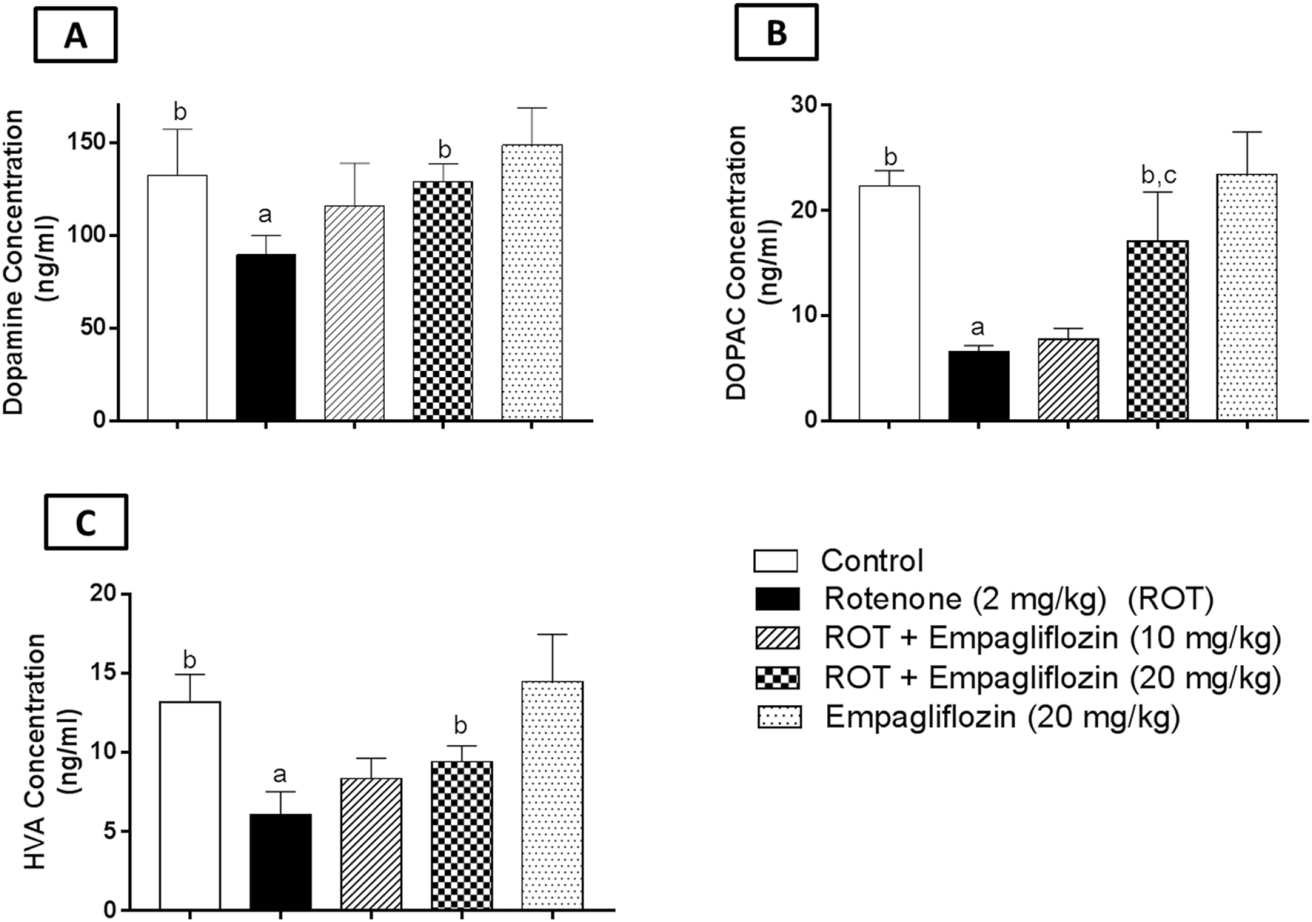
Effect of Treatment with EMPA on the midbrain and striatal dopamine, DOPAC, and HVA level in an experimental model of PD induced by rotenone. Data are presented as mean ± SD (*n* = 6), where a, b, and c are statistically significant from control, rotenone-treated group, and EMPA (10 mg/kg)-treated groups, respectively, at *p* < 0.05 using one-way analysis of variance (ANOVA) followed by Tukey as a post-hoc test

The striatal concentration of DA (F_4,25_ = 8.381, *p* = 0.0002) and its metabolites, DOPAC (F_4,25_ = 44.87, *p* < 0.0001), and HVA (F_4,25_ = 21.81, *p* < 0.0001) were detected using HPLC for DA turnover assessment (Fig. 4). The concentration of DA (Fig. 4A) and its metabolites; DOPAC (Fig. 4B) and HVA (Fig. 4C) were found to be a significantly lower in the rotenone-treated group by 1.48, 3.4, and 2.195 folds, respectively, relative to the corresponding control group. Conversely, rats treated with EMPA at a dose of 20 mg/kg revealed a significant elevation in DA, DOPAC, and HVA concentrations by 1.44, 2.6, and 1.56 folds when compared to rotenone-treated rats. Additionally, the higher dose of EMPA illustrated a significant elevation in DOPAC concentration by 2.22 folds compared to the lower dose.

### Effect of EMPA treatment on alpha-synuclein and TH expression in rotenone-induced PD in rats

**Fig. 5:**
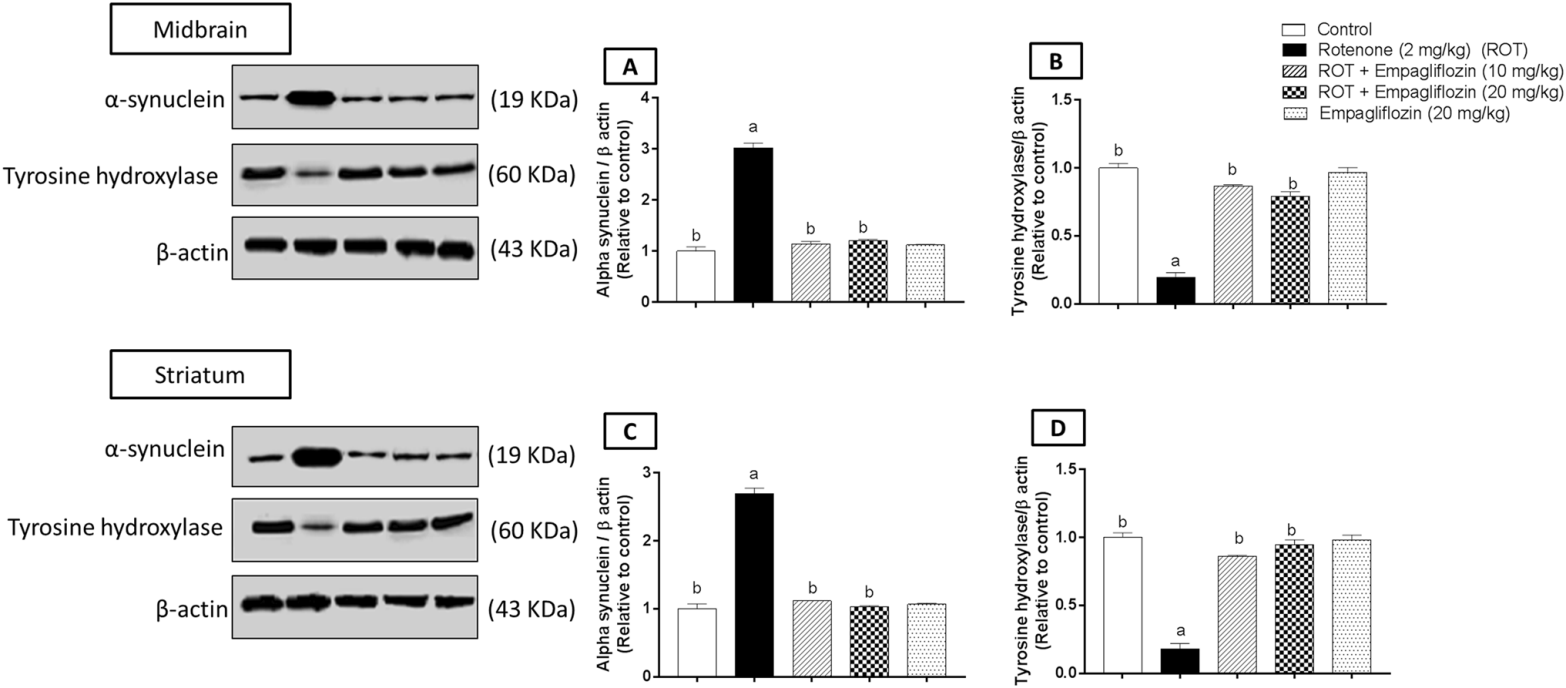
Effect of Treatment with EMPA on the midbrain and the striatum expression of α-synuclein and TH activity in an experimental model of PD induced by rotenone. Representative photomicrographs for the control group (**A**); rotenone-treated group (2 mg/kg) (**B**); rotenone and EMPA group (10 mg/kg) (**C**); rotenone and EMPA group (20 mg/kg) (**D**); EMPA alone group (20 mg/kg) (**E**); Data are presented as mean ± SD (*n* = 3) where: a and b; statistically significant from the control group and rotenone-treated groups, respectively, at *p* < 0.05 using one-way analysis of variance (ANOVA) followed by Tukey as a posthoc test

Concerning alpha-synuclein expression in the midbrain region **(**Fig. 5A**)**, rotenone-treated rats revealed enhancement in the expression of alpha-synuclein by 3.025 folds relative to the control group (F_4,10_ = 581.7, *p* < 0.0001). By contrast, Treatment with EMPA, 10 mg/kg and 20 mg/kg, significantly reversed this elevation by 2.64 and 2.49 folds, respectively, compared to rotenone-treated rats. Regarding TH expression in the midbrain (Fig. 5B), rotenone treatment exhibited a significant reduction in TH expression by 5.13 folds relative to the control group (F_4,10_ = 340.0, *p* < 0.0001). However, EMPA (10 mg/kg) treated rats illustrated a significant elevation in TH expression by 4.45 folds compared to the rotenone-treated group. Higher EMPA dose treatment revealed a 4.05-fold significant elevation compared to the rotenone-treated group.

Regarding alpha-synuclein expression in the striatum (Fig. 5C), rotenone treatment showed a significant increase in alpha-synuclein expression by 2.69 folds compared to the negative control group. In comparison, a significant decrease by 2.33 folds was detected when comparing the EMPA (10 mg/kg)-treated group with the rotenone-treated group (F_4,10_ = 641.1, *p* < 0.0001). A 2.62-fold significant reduction was found in EMPA (20 mg/kg)-treated rats relative to the rotenone-treated group. Concerning TH expression in the striatum region (Fig. 5D), there was a significant reduction by 5.52 folds in the rotenone-treated group as compared to the control group (F_4,10_ = 345.6, *p* < 0.0001). In contrast, there was a significant elevation following 10 mg/kg and 20 mg/kg EMPA treatment as compared to the rotenone-induced group by 4.77 and 5.24 folds, respectively.

### Effect of EMPA treatment on oxidative stress markers in rotenone-induced PD in rats

**Fig. 6:**
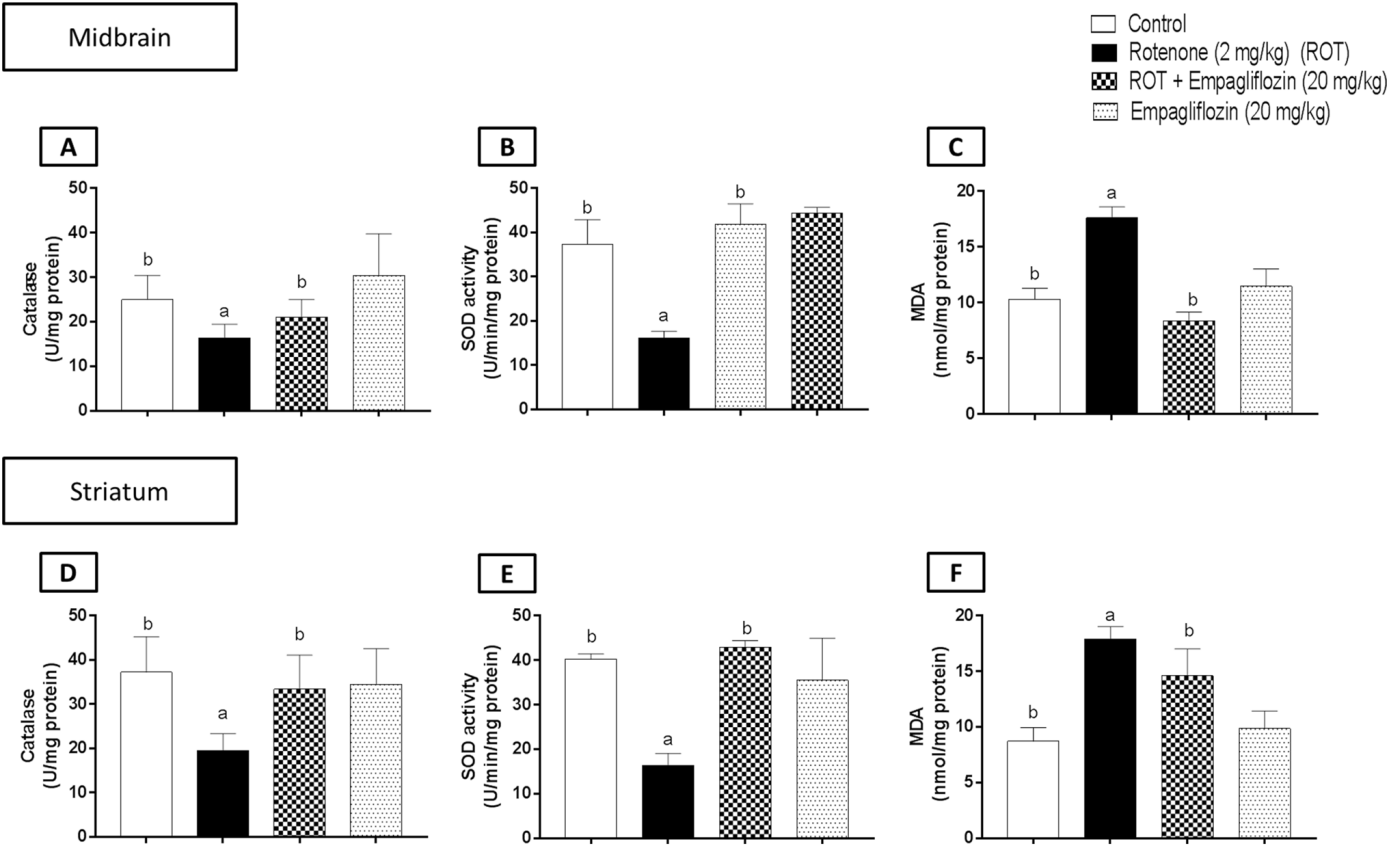
Effects of EMPA on the midbrain and striatal levels of catalase (**A**), superoxide dismutase (**B**), and malondialdehyde (**C**) in rotenone-induced PD in rats. Data are presented as means ± S.D. (*n* = 6). Where: a and b; statistically significant from the control group and rotenone-treated groups, respectively, at *p* < 0.05 using one-way analysis of variance (ANOVA) followed by Tukey as a posthoc test

To assess the effect of EMPA treatment on oxidative stress markers in rotenone-induced PD in rats, antioxidant CAT enzyme, lipid peroxidation MDA, and SOD activity were detected in the midbrains and striata of different groups (Fig. 6).

First, rotenone treatment significantly reduced the midbrain CAT level by 1.62 folds relative to the control group (F_3,20_ = 7.654, *p* = 0.0013). EMPA (20 mg/kg) treated group illustrated a significant elevation in catalase activity by 1.59 folds compared to the disease group (Fig. 6A). Rotenone-treated group revealed a significant reduction in catalase level in the striatum relative to the control rats by 1.91 folds (F_3,20_ = 7.425, *p* = 0.0016). Treatment with 20 mg/kg EMPA showed a significant increase in striatal catalase level relative to rotenone-treated rats by 1.71 folds (Fig. 6D).

Moreover, rotenone treatment illustrated a significant reduction in SOD activity in both the midbrain (Fig. 6B) (F_3,20_ = 70.3, *p* < 0.0001) and the striatum (Fig. 6E) (F_3,20_ = 34.47, *p* < 0.0001) by 2.3 and 2.46 folds, respectively, relative to the corresponding control group. On the other hand, the EMPA-treated group exhibited a significant increase in the midbrain and striatal SOD activity by 2.75 and 2.63 folds, respectively.

Furthermore, the rotenone-treated group revealed a significant elevation in MDA level in both the midbrain (Fig. 6C) (F_3,20_ = 75.84, *p* < 0.0001) and the striatum (Fig. 6F) (F_3,20_ = 39.07, *p* < 0.0001) by 1.71 and 2.05 folds, respectively, compared to the corresponding control group. However, Treatment with EMPA showed a significant reduction in lipid peroxidation levels in both the midbrain and the striatum by 2.1 and 1.22 folds, respectively.

### Effect of EMPA treatment on inflammatory markers in rotenone-induced PD in rats

**Fig. 7:**
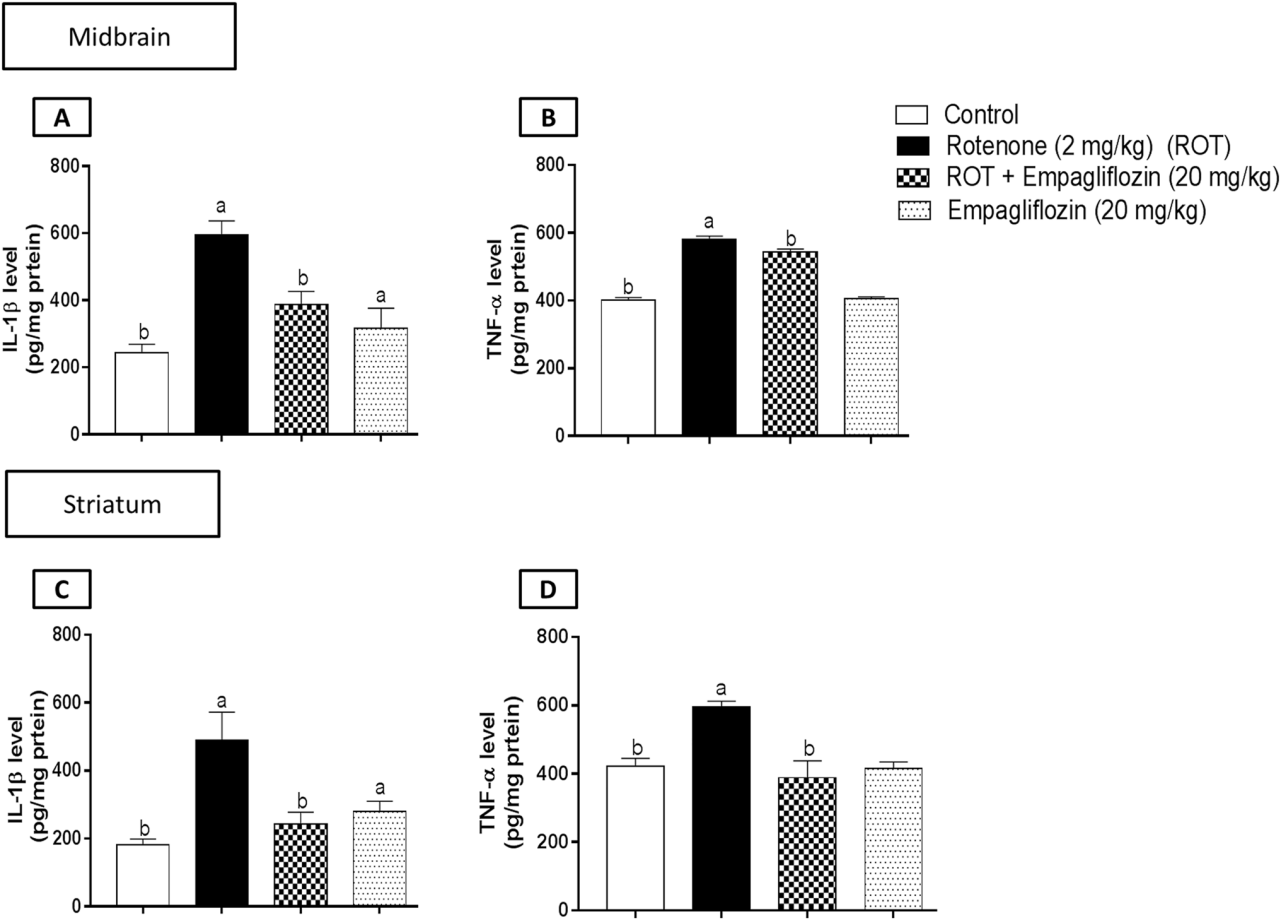
Effect of EMPA treatment on the midbrain IL-1β (**A**), the midbrain TNF-α (**B**), the striatum IL-1β (**C**), the striatum TNF-α (**D**) levels in an experimental model of PD induced by rotenone. Data are presented as means ± SD (*n* = 6) and analyzed by one-way ANOVA followed by Tukey posthoc test where: a and b indicate statistically significant from control and rotenone-treated groups, respectively, at *p* < 0.05

Levels of TNF-α and IL-1β were determined for assessment of anti-inflammatory effects of EMPA (Fig. 7). Concerning IL-1β, rotenone-treated group illustrated a significant elevation in both the midbrain (Fig. 7A) (F_3,20_ = 72.09, *p* < 0.0001) and striatal (Fig. 7C) (F_3,20_ = 46.46, *p* < 0.0001) IL-1 β compared to negative control group by 2.44 and 2.69 folds, respectively. By contrast, the group treated with EMPA reversed such elevation in a significant manner relative to the rotenone-treated group by 1.53 folds in the midbrain and 2.02 folds in the striatum. On comparing negative control and drug-alone groups, there were 1.29 folds of a significant elevation in the midbrain and 1.54 folds in the striatum.

Assessment of TNF-α level showed a significant decrease in the midbrain (Fig. 7B) (F_3,20_ = 825.6, *p* < 0.0001) and the striatum (Fig. 7D) (F_3,20_ = 58.5, *p *< 0.0001) of the rotenone-treated group by 1.45 and 1.41 folds, respectively, compared to the corresponding control group. However, the EMPA (20 mg/kg) treated group showed a significant reduction in the midbrain TNF-α level by 1.07 folds and striatal TNF-α level by 1.53 folds compared to the rotenone-treated group.

### Effect of EMPA treatment on expression of AMPK, pAMPK, SIRT-1, PGC-1α, and β-catenin in rotenone-induced PD in rats.

**Fig. 8:**
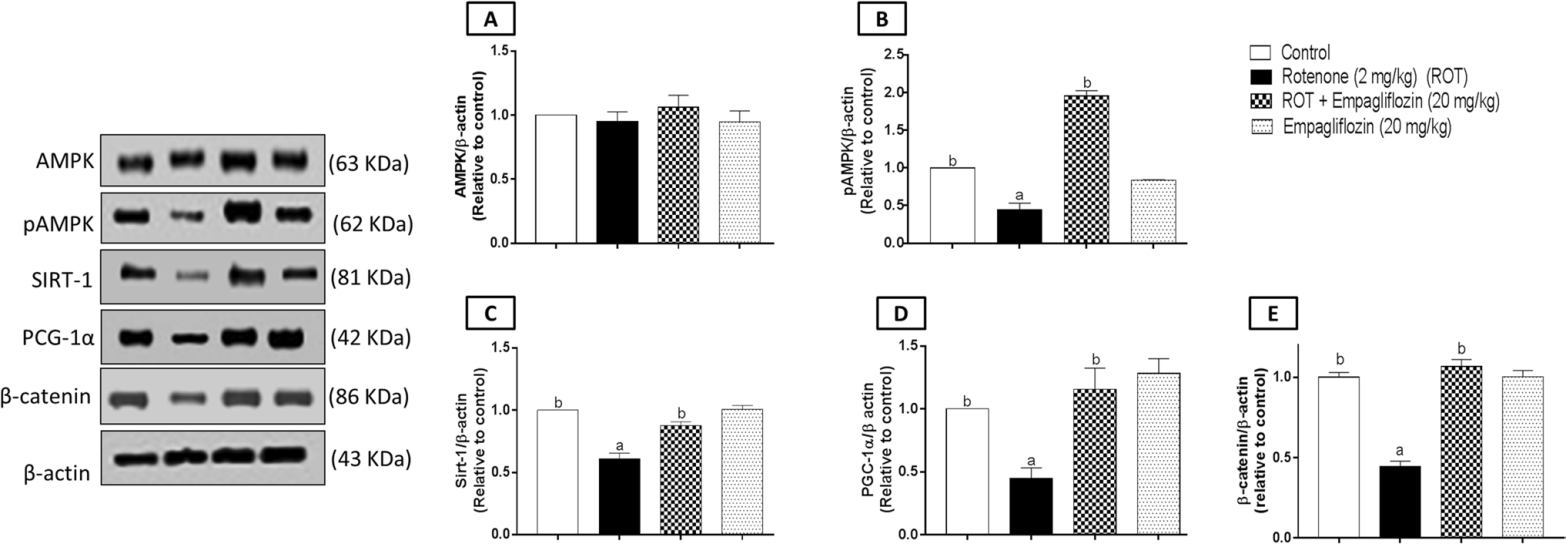
Western blot analysis of the midbrain AMPK (**A**), pAMPK (**B**), SIRT-1 (**C**), PGC1-α (**D**), and β-catenin (**E**) for the control group, rotenone-treated group, rotenone and EMPA (20 mg/kg) group, and EMPA (20 mg/kg) alone group. Data are presented as means ± SD (*n* = 3) and analyzed by one-way ANOVA followed by Tukey post hoc test where: a and b indicate statistically significant from the control and rotenone-treated groups, respectively, at *p* < 0.05

**Fig. 9:**
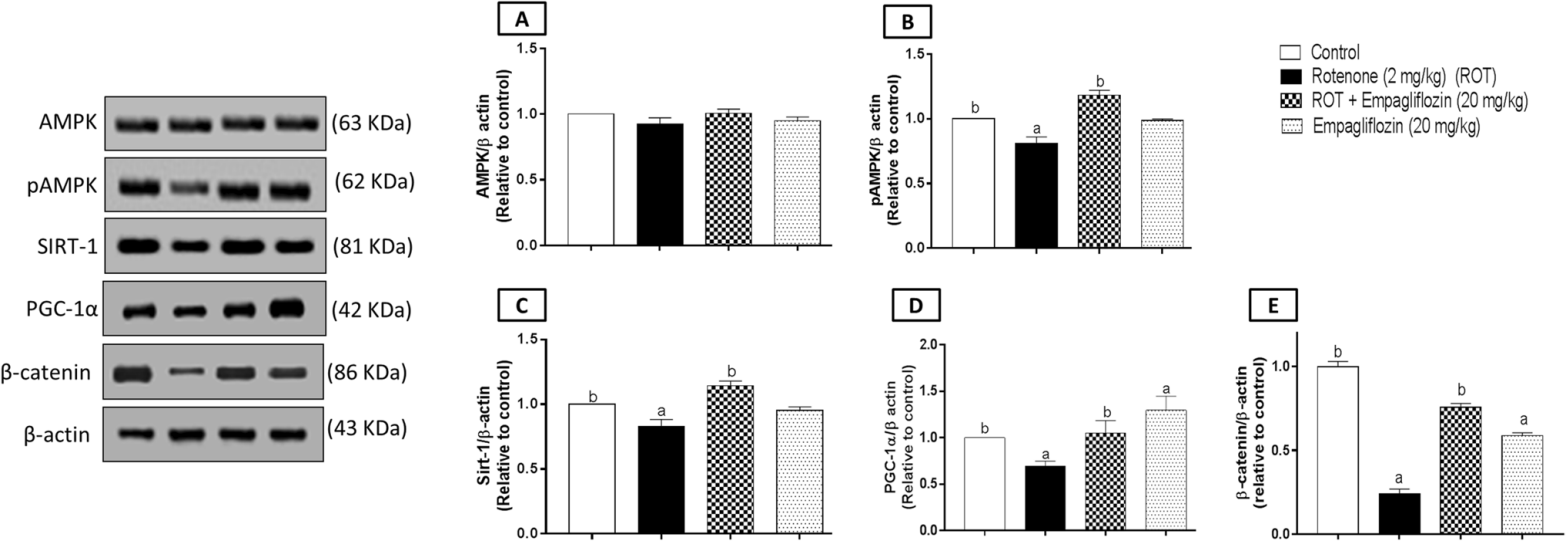
Western blot analysis of the striatum AMPK (**A**), pAMPK (**B**), SIRT-1 (**C**), PGC1-α (**D**), and β-catenin (**E**) for the control group, rotenone-treated group, rotenone and EMPA (20 mg/kg) group, and EMPA (20 mg/kg) alone group. Data are presented as means ± SD (*n* = 3) and analyzed by one-way ANOVA followed by Tukey post hoc test where: a and b indicate statistically significant from the control and rotenone-treated groups, respectively, at *p* < 0.05

One-way ANOVA revealed no significant difference between different groups concerning AMPK expression in both the midbrain (Fig. 8A) (F_3,8_ = 1.603, *p* = 0.2636) and the striatum (Fig. 9A) (F_3,8_ = 4.691, *p* = 0.0358).

Regarding the midbrain, the expression of pAMPK (Fig. 8B) in rotenone treatment illustrated a significant decrease compared to the control group by 2.23 folds (F_3,8_ = 422.4, *p* < 0.0001). However, a significant increase was detected by 1.957 folds relative to the higher dose of EMPA compared to the rotenone-induced group. Moreover, a 1.63 significant reduction was found concerning SIRT-1 expression (Fig. 8C) when comparing the PD-induced group with the control group. In contrast, a significant elevation was detected by 1.43 folds on comparing the rotenone-induced group with the group treated with 20 mg/kg EMPA (F_3,8_ = 101.5, *p* < 0.0001). Concerning PGC-1α expression (Fig. 8D), a significant reduction was detected in the disease group by 2.23 folds compared to the control group, while 2.58 folds of a significant elevation were determined compared to the higher dose of EMPA with the disease group (F_3,8_ = 33.38, *p* < 0.0001).

Concerning the striatum, there was a significant decrease in pAMPK expression (Fig. 9B) by 1.23 folds in the disease group compared to the control group (F_3,8_ = 69.62, *p* < 0.0001). A significant elevation was detected by 1.46 folds revealed to the disease group. In addition, a significant decrease in the control group was revealed to the drug-alone group by 1.45 folds. In addition, a significant decrease was detected in SIRT-1 expression (Fig. 9C) by 1.2 folds compared with the control group (F_3,8_ = 43.73, *p* < 0.0001). In contrast, a significant elevation was 1.375 folds in SIRT-1 expression when comparing the rotenone-induced group with those treated with 20 mg/kg EMPA. Moreover, a significant decrease was detected on assessment of PGC-1α expression (Fig. 9D) by 1.44 folds in the rotenone group compared to the control group, while the drug alone group showed a significant elevation by 1.51 folds compared with the control group (F_3,8_ = 16.54, *p* = 0.0009).

For β-catenin determination, a significant decrease was shown in the midbrain (Fig. 8E) of the disease group by 2.25 and 2.4 folds compared to the control group and EMPA-treated group, respectively (F_3,8_ = 186.5, *p* < 0.0001). In addition, a significant reduction was shown in the striata (Fig. 9E) of the disease group by 4.12 and 3.12 folds relative to the negative control group and EMPA (20 mg/kg), respectively (F_3,8_ = 486.3, *p* < 0.0001). The drug-alone-treated group revealed a significant reduction in striatal β-catenin expression compared to the control group by 1.69 folds.

### Effect of EMPA treatment on NAD^+^/NADH and wnt-3a levels in rotenone-induced PD in rats.

**Fig. 10:**
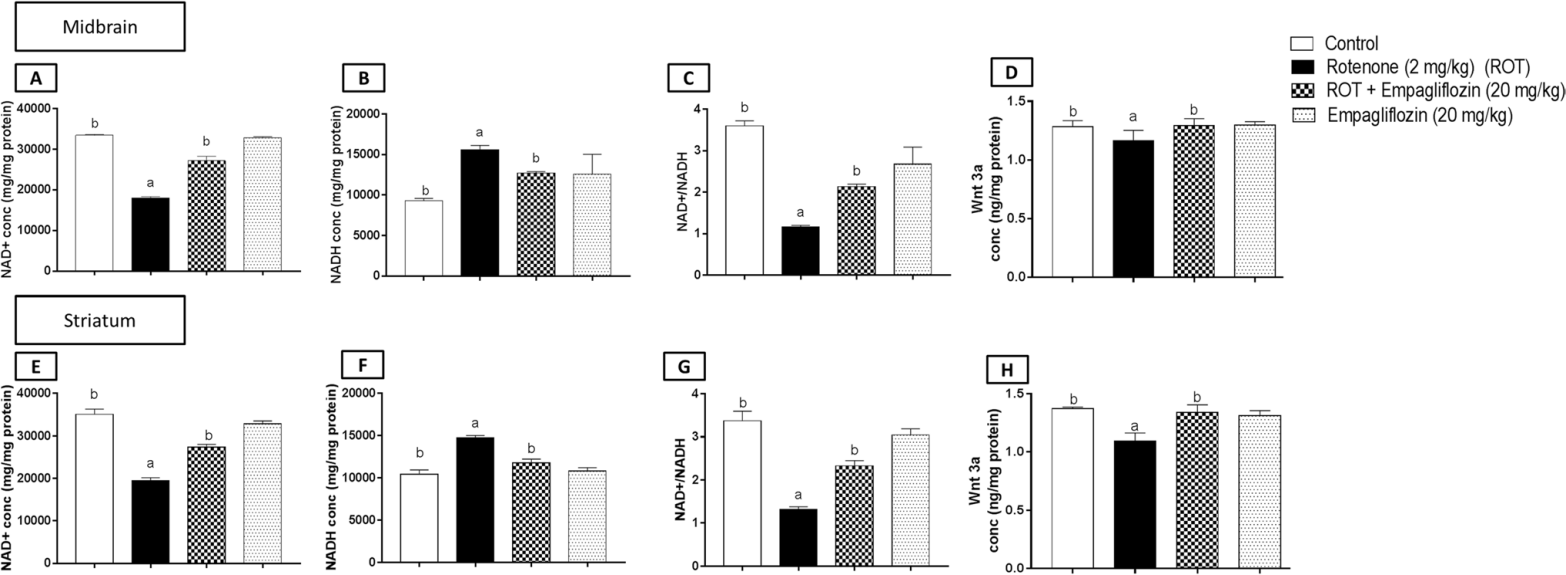
Effect of EMPA treatment on NAD.^+^, NADH, NAD/NADH, and wnt 3a levels in an experimental model of PD in rats. Data are presented as means ± SD (*n* = 6) and analyzed by one-way ANOVA followed by Tukey post hoc test where: a and b indicate statistically significant from the control and rotenone-treated groups, respectively, at *p* < 0.05

The NAD + , NADH, NAD + /NADH ratio, and wnt-3a levels were determined in both the midbrain and the striatum (Fig. 10).

Concerning NAD^+^ assessment in the midbrain (Fig. 10A), the rotenone-treated group revealed a significant reduction in NAD^+^ level by 1.85 folds, while a significant elevation was detected for the same area in the higher dose-treated group of EMPA by 1.51 folds compared to the disease group (F_3,20_ = 869.2, *p* < 0.0001). However, in the striatum, the rotenone group showed a significant decrease by 1.8 folds compared to the control group (F_3,20_ = 404.7, *p* < 0.0001). Moreover, a 1.4-fold significant increase was detected compared to the group treated with 20 mg/kg EMPA with the disease group. Additionally, the drug-alone group revealed a significant reduction by 1.06 folds relative to the control group (Fig. 10E).

Regarding NADH assessment, there was a significant elevation in the rotenone group compared to the negative control group in the midbrain (Fig. 10B) (F_3,20_ = 24.45, *p* < 0.0001) and the striatum **(**Fig. 10F**)** (F_3,20_ = 128.1, *p* < 0.0001) by 1.76 and 1.41 folds, respectively. In contrast, the drug-alone group showed a significant increase by 1.35 folds relative to the control group. Additionally, the higher dose of EMPA showed a significant reduction by 1.25 folds compared to rotenone-treated rats.

Concerning NAD^+^/NADH ratio, the rotenone-treated group revealed a significant decrease compared to the negative control group in the midbrain (Fig. 10C) (F_3,20_ = 133.7, *p* < 0.0001) and the striatum (Fig. 10G) (F_3,20_ = 221.9, *p* < 0.0001) by 3.10 and 2.54 folds, respectively. Besides, a significant elevation was determined in the EMPA-treated group of rats by 1.84 folds in the midbrain and by 1.8 folds in the striatum relative to the corresponding rotenone-treated group.

Concerning wnt-3a assessment, a significant reduction was detected in the disease group by 1.1 and 1.25 folds on comparison with the control group in the midbrain (Fig. 10D) (F_3,20_ = 6.732, *p* = 0.0025) and the striatum (Fig. 10H) (F_3,20_ = 33.55, *p* < 0.0001), respectively. However, the EMPA (20 mg/kg) treated group significantly increased by 1.11 and 1.23 folds compared to the midbrain and striatum disease groups, respectively.

## Discussion

PD is characterized by the progressive loss of dopaminergic neurons in the substantia nigra pars compacta, as well as alpha-synuclein and Lewy bodies aggregation (Zheng et al. 2021). PD was reported to be associated with exacerbation of oxidative stress, inflammatory response, and energy metabolism homeostasis disturbance (Kaur et al. 2021). Rotenone was reported to induce oxidative stress, neuroinflammation, and accumulation of alpha-synuclein, which recapitulates many of PD's behavioral, neurochemical, and neuropathological features (Buck et al. 2021). EMPA was reported previously for its possible neuroprotective effects (Pawlos et al. 2021). Hence, the present study aimed to evaluate the potential neuromodulatory effects of EMPA on rotenone-induced PD in rats and identify the possible underlying mechanisms.

Rotenone, actually used as a fat-soluble insecticide, is a high-affinity inhibitor of the mitochondrial complex-I and oxidative stress inducer, leading to the death of dopaminergic cells. Therefore, rotenone seems to propagate almost all the hallmarkers of PD, including the accumulation of α-synuclein and the formation of Lewy bodies (Siracusa et al. 2020). Rotenone administration initiates a neurodegenerative cascade leading to a spontaneous progression of parkinsonian phenotype. In this context, rotenone was administrated for 30 consecutive days, a delayed progressive motor and behavior disorders was observed after few days. As the motor symptoms have proceeded, there was an evidence of ongoing degeneration of striata and consequently neuroinflammation, restating similar pathological and motor attribution of the human disease (Van Laar et al. 2023). Over the ensuing of that month, there was progressive microglial activation and aggregation of α-synuclein in substantia nigra. In addition to, neuropathological features have progressed, focusing on rotenone’s ability to initiate the two pathological hallmarks of PD, motor deficits, pathological condition of PD involvement at clinically relevant time points (Johnson and Bobrovskaya 2015). Therefore, progression of PD-like symptoms and neurological features of PD following rotenone administration were found to mimic the progression of PD in human patients.

In the present study, the doses used for EMPA; 10 and 20 mg/kg, are equivalently to the clinical doses approved by US Food and Drug Administration (FDA) for the treatment of patients with Type II diabetes. These doses were selected to mimic EMPA doses used in humans. Moreover, the availability of both doses in the market promotes the practical use possibility in clinical trials. Additionally, these doses were reported by regulatory agencies to be safe and effective. This may encourage the clinical use of EMPA with the above-mentioned doses and may enter clinical trials for management of PD (Fala 2015).

A preliminary dose–response study was performed to select the effective EMPA dose. Rats were divided into five groups: control group, rotenone-treated group, rotenone + EMPA (10 mg/kg)-treated, rotenone + EMPA (20 mg/kg)-treated, and EMPA (20 mg/kg)-treated groups. Afterward, behavioral tests, histological examination, intact neuronal staining, alpha-synuclein and TH expression, and DA, DOPAC, and HVA levels were detected.

Behavioral assessment was established to determine motor impairment in the PD model. Rotenone treatment caused a decline in locomotor activity, elongation of descent latency time in catalepsy tests, and reduced retention time on practicing rotarod performance tests. This may indicate rigidity and motor dysfunction mimicking PD behavioral changes. These results were in agreement with Prasad and Hung (2020). On the other hand, both doses of EMPA (10 and 20 mg/kg) corrected such motor impairment in the tests mentioned above. This may conclude the positive impact of EMPA treatment on neurobehavioral changes associated with PD. It is worth mentioning that a higher dose of EMPA (20 mg/kg) revealed a superior effect relative to a lower dose in both locomotor activity and rotarod tests.

Behavioral tests were further confirmed by histological examination. Rotenone-induced brain defects were proven by histological staining of the midbrain, substantia nigra, and the striatum areas. Microscopic examination of the above areas revealed severe neuronal loss and moderate edema of the brain matrix accompanied by many reactive glial cell infiltrates. These results were convenient with the results of Swarnkar et al. (2011). On the other hand, EMPA, at a dose of 10 mg/kg, ameliorated the number of degenerated neurons and showed significant neuroprotective efficacy with mild edema and microglial cell activation. Interestingly, the higher dose of EMPA demonstrated more protective effects with nearly no abnormal histological changes.

Since PD is always associated with neurodegeneration, the three stained areas, the midbrain, substantia nigra, and the striatum, were stained by nissl stain to assess rotenone and EMPA effect on number of intact neurons. Rotenone-treated rats revealed a significant elevation in the number of degenerated neurons, supported by the results of Salama et al. (2020). Treatment with EMPA at both doses, 10 and 20 mg/kg corrected such neurodegenerative effects in the three areas. Interestingly, EMPA (20 mg/kg) showed more significant neuronal degeneration reduction in the striatum and substantia nigra relative to lower dose (10 mg/kg) treatment.

The primary neurotransmitter that was reported to be affected in PD is DA. Different pathways into DOPAC and HVA normally metabolize DA. Previous research observed that PD is linked with reduced DA content (Lyng and Seegal 2008). Dopamine neuron degeneration is associated with a remarkable decrease in DA levels. DOPAC and HVA concentrations were assessed to indicate the neuronal dopaminergic activity (Kremer et al. 2021). The results of the recent study demonstrated that rotenone-treated rats showed a significant reduction in striatal DA, DOPAC, and HVA content. On the contrary, EMPA (10 mg/kg and 20 mg/kg) reversed and increased the concentrations of DA and its metabolites. Interestingly, EMPA of dose (20 mg/kg) showed a more significant increase in DOPAC concentration than EMPA (10 mg/kg).

The assessment of TH supported the previous results, the rate-limiting enzyme of the DA biosynthesis pathway, as it induces the formation of L-DOPA, which is the rate-limiting step in DA synthesis. Moreover, phosphorylation and reduction of TH indicate dopamine loss compensation (Kawahata and Kohji 2020). Previous studies reported that directly increasing TH activity and elevated dopamine turnover are critical in oxidative stress as a source of H_2_O_2_ and other reactive oxygen species (Vecchio et al. 2021). In our study, rotenone-treated rats showed a significant reduction in TH expression aligned with the study (Abdel-Salam et al. 2015). By contrast, both EMPA doses (10 mg/kg and 20 mg/kg) showed a significant elevation in TH expression in the midbrain and the striatum; these results were supported by the study of Castoldi et al. (2021).

A classical hallmark for PD is the accumulation of α-synuclein (Eriksen et al. 2003). It was reported that alpha-synuclein accumulation might impair normal cellular functions and promote oxidative stress. Our results showed that rotenone treatment augmented alpha-synuclein expression in both the midbrain and the striatum areas. This was following the study of Sala et al. (2013). On the contrary, EMPA treatment reversed this elevation in both study areas, decreasing the accumulated alpha-synuclein level.

Depending on the results obtained from the preliminary dose–response study, the higher dose of EMPA (20 mg/kg) was selected for further mechanistic studies.

Oxidative stress is the inability of the biological system to maintain redox balance, resulting in an imbalance between ROS production and elimination. A significant association was observed between PD and oxidative stress. Mitochondrial dysfunction, neuroinflammation, DA metabolism, and misfolded alpha-synuclein aggregates may promote PD oxidative stress (Chang and Chiung-Mei 2020). Therefore, oxidative stress-related signaling pathways could be a therapeutic target to prevent oxidative stress generation in PD patients (Khan and Sharique 2018). Rotenone crosses the blood–brain barrier to reach and inactivate the mitochondrial complex I, leading to reduced ATP production, ROS elevation, and dopaminergic neuron loss (Blesa et al. 2015). In this context, CAT, MDA, and SOD activity were assessed in our study for oxidative stress evaluation. Our results revealed that rotenone treatment decreased CAT and SOD activity and enhanced MDA levels. Such effects were recorded for both the midbrain and the striatum. EMPA treatment caused reverts in the antioxidant CAT and SOD activities and oxidative MDA levels in the midbrain and the striatum (Motawi et al. 2022).

Besides, neuroinflammation was reported to contribute significantly to PD progression. Accumulation of degenerated dopaminergic neurons causes innate and adaptive immune response activation (Fathi et al. 2022). In addition, activated microglial cells by alpha-synuclein induce the release of proinflammatory cytokines, IL-1β and TNF-α, leading to neuroinflammation and neurodegeneration (Tansey et al. 2022; Yang et al. 2008). Conversely, neuroinflammation may lead to the loss of dopaminergic neurons, precipitating the progression of neurodegeneration in PD (Ramirez et al. 2017). IL-1β is a type of IL-1 proinflammatory cytokines linked with permanent and irreversible dopaminergic neuronal loss in substania nigra. The effect of IL-1β on dopaminergic cell loss depends on its concentration, expression duration, and stimuli timing (Stojakovic et al. 2017). Rotenone elevated the expression levels of IL-1β due to microglial activation, which was supported by postmortem studies (Sharma et al. 2018). TNF-α is an important proinflammatory cytokine belonging to TNF family-induced genes regulating inflammation and proliferation (Hayden and Sankar 2014). Moreover, elevated levels of TNF-α were observed in the substantia nigra of patients with PD. Hence, TNF-α plays a vital role in the degeneration of DA neurons by inflammatory responses mediated by the microglia (Erekat and Muhammed 2018).

Expectedly, our results showed that rotenone elevated IL-1β and TNF-α levels in the midbrain and the striatum. Such results were reported previously by Javed et al. (2016). EMPA treatment could halt the effect of rotenone on inflammatory cytokines, evidenced by its significant effect on TNF-α and IL-1β levels in both studied areas. These results were supported by the results of Ahmed et al. (2022).

Energy metabolism regulation was recently disturbed in the PD, AMPK/SIRT-1/PGC-1α pathway, which was reported to regulate energy homeostasis. AMPK and SIRT-1 are essential energy regulatory factors that regulate glucose homeostasis and stimulate mitochondrial biogenesis. Additionally, it was proven that activation of the AMPK/SIRT-1/PGC-1α pathway is a potential candidate for the Treatment of PD (Jhuo et al. 2020). Reduction of ATP levels during energy depletion stimulates AMPK phosphorylation and its activation. The latter activates SIRT-1 as a compensatory mechanism for energy depletion. PGC-1α is the master regulator of mitochondrial biosynthesis, regulating mitochondrial function and cellular energy metabolism, and acts as a transcriptional co-activator (McMeekin et al. 2021). PGC-1α should undergo phosphorylation and deacetylation to be active. PGC-1α phosphorylation could be mediated via AMPK (George et al. 2023). During PD-associated neurodegeneration, lowered ATP levels induce AMP accumulation and AMPK activation. Activated (phosphorylated) AMPK promotes PGC-1α phosphorylation using NAD^+^ as a co-factor.

Additionally, AMPK activates SIRT-1, which could deacetylate PGC-1α, activating it. Piccinin et al. (2021) revealed that sufficient PGC-1α levels provide proper neuronal functions. Moreover, maintaining the level of SIRT-1 and PGC-1α induced protection against oxidative stress and improved PD symptoms via reduced cytokine levels (Corona and Duchen 2015; Li et al. 2020).

In our study, rotenone treatment ameliorated AMPK phosphorylation in both the midbrain and striata, significantly reducing SIRT-1 and PGC-1α expression. However, the treatment of EMPA (20 mg/kg) opposed the effect of rotenone elevating the expression of pAMPK, SIRT-1, and PGC-1α in both studied brain areas. Our observations were supported by the study by Kim et al. (2019). Additionally, researchers proved the importance of NAD^+^ for restoring mitochondrial function, leading to improved energy homeostasis (Lautrup et al. 2019; Fang et al. 2016). In our study, it was observed that rotenone treatment caused a reduction in NAD^+^ level, resulting in a reduction of AMPK activation and SIRT-1 deacetylation. On the other hand, opposite results were observed for NADH following rotenone treatment. However, EMPA treatment restored the NAD + NADH levels and their ratio. Therefore, EMPA treatment may exert its neuromodulatory effects in PD via activating the AMPK/SIRT-1/PGC-1α axis.

The canonical wnt pathway was reported for its role in proliferation, cell survival, and neurodevelopment (Ng et al. 2019). In addition, researchers proved that the wnt/β-catenin pathway has a vital role in dopaminergic neurogenesis for PD patients (Marchetti et al. 2020). Wnt-3a ligand binds to its cell surface receptor (Frizzled), which phosphorylates the LRP 5/6 subunit, promoting recruitment of accessible glycogen synthase kinase-3β (GSK-3β) and Axin. This prevents the formation of the destructive complex of APC/CK1/AXIN/GSK-3β, setting β-catenin free. The latter acts as a transcription factor for TCF/LEF genes inside the nucleus for neurodevelopment (George et al. 2020). However, oxidative stress and neuroinflammation could inhibit wnt-3a binding to its receptor, turning the wnt/β-catenin pathway off. In this case, the destructive complex is formed by inducing β-catenin phosphorylation and degradation via proteasomes (L'Episcopo et al. 2018).

In our study, rotenone administration is associated with dysregulation of wnt signaling via hindering wnt-3a level and reduction of β-catenin expression in both the midbrain and striatum, which agreed with the results of Stephano et al. (2018). On the contrary, EMPA treatment induced a significant elevation in wnt-3a level and β-catenin expression, giving sufficient evidence for stimulation of the wnt/β-catenin pathway. The study of Cai et al. (2022) found that EMPA treatment could induce wnt/β-catenin pathway activation.

## Conclusion

Our study showed that rotenone administration resulted in dopaminergic neuron degeneration, alpha-synuclein expression enhancement, and reduction of tyrosine hydroxylase expression. Moreover, rotenone administration induced oxidative stress and inflammatory response in the striatum and the midbrain. Additionally, after rotenone treatment, the amelioration of AMPK/SIRT-1/PGC-1α and inhibition of the wnt/β-catenin pathway were ameliorated. By contrast, EMPA administration reversed rotenone's effects, as evidenced by modulating motor activity, histopathological alterations, dopamine turnover, and alpha-synuclein expression level. Additionally, EMPA corrected redox imbalance and inflammatory response induced by rotenone via its antioxidant and anti-inflammatory activities. Via activation of AMPK/SIRT-1/PGC-1α and canonical wnt/β-catenin pathways, EMPA could induce its beneficial neuromodulatory effects in PD.

## Data Availability

Data will be made available on reasonable request.
